# Diffuse anterior and posterior scleritis with multiple iris granular deposits following pterygium excision

**DOI:** 10.22336/rjo.2021.79

**Published:** 2021

**Authors:** Kazuki Matsuura, Yuki Terasaka

**Affiliations:** * Nojima Hospital, Tottori, Japan

**Keywords:** pterygium surgery, surgically induced necrotizing scleritis (SINS), posterior scleritis

## Abstract

Surgically induced necrotizing scleritis has been reported after several types of ophthalmic surgeries; however, not many cases are reported following pterygium surgery (PS).

A 79-year-old woman underwent primary pterygium excision and conjunctival autograft transportation with mitomycin C in her left eye. 18 months postoperatively, diffuse anterior and posterior scleritis was noted; however, scleral necrosis was not apparent. Multiple granular deposits were observed on the surface of the iris. The deposits, aqueous humor, and vitreous were examined. Since there were no signs of infection or malignancy, the patient was diagnosed with scleritis with intraocular inflammation following PS.

Necrosis was accompanying at the surgical site in most cases of scleritis following PS. However, the scleral necrosis of the surgical site was not significant in our case. Posterior scleritis associated with PS has never been reported. This is the first report of anterior diffuse scleritis accompanied by posterior scleritis following PS.

**Abbreviations:** PS = pterygium surgery, SINS = surgically induced necrotizing scleritis, MMC = mitomycin C, ANCA = antineutrophil cytoplasmic antibody

## Introduction

Surgically induced necrotizing sclerokeratitis (SINS) has been reported following several types of ophthalmic surgeries such as strabismus, retinal detachment, cataract, and glaucoma surgery [**1**-**3**]. However, only a few cases have been reported after primary pterygium excision. To the best of our knowledge, posterior scleritis associated with pterygium surgery has not been reported [**4**-**8**]. We reported such a case of anterior diffuse scleritis accompanied with posterior scleritis, following primary pterygium excision.

## Case report

**Fig. 1:**
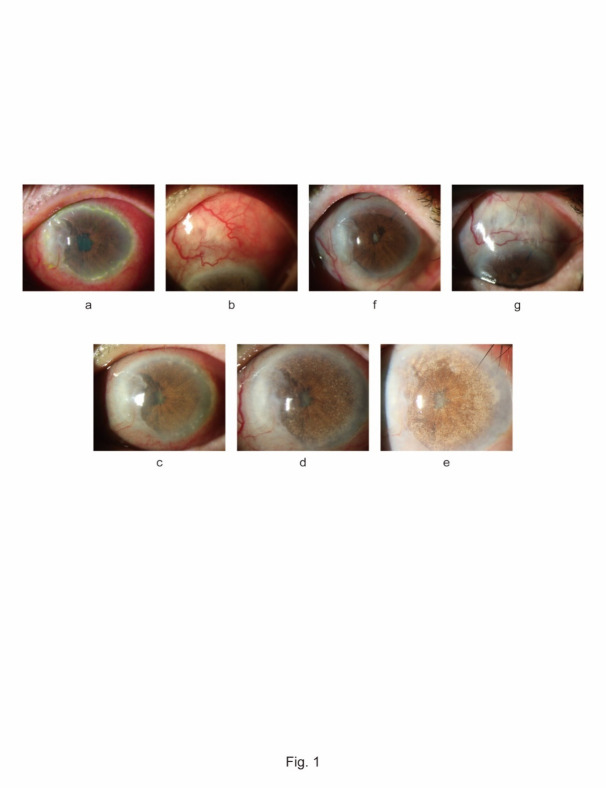
Photograph of the anterior segment of the left eye. **Fig. 1a, b** Scleral edema and dilation of the deep episcleral vascular plexus with infiltrates of peripheral cornea and slight thinning of the adjacent cornea to the excision site; **Fig. 1c-e** Multiple deposit granules were visible on the iris. The deposit gradually increased over the next 4 weeks, and some of them were stripped from the iris and floated in the anterior chamber; **Fig. 1f, g** Decreased scleral edema and dilation of the deep episcleral vascular plexus after treatment for anterior diffuse scleritis. Iris deposits almost disappeared

**Fig. 2:**
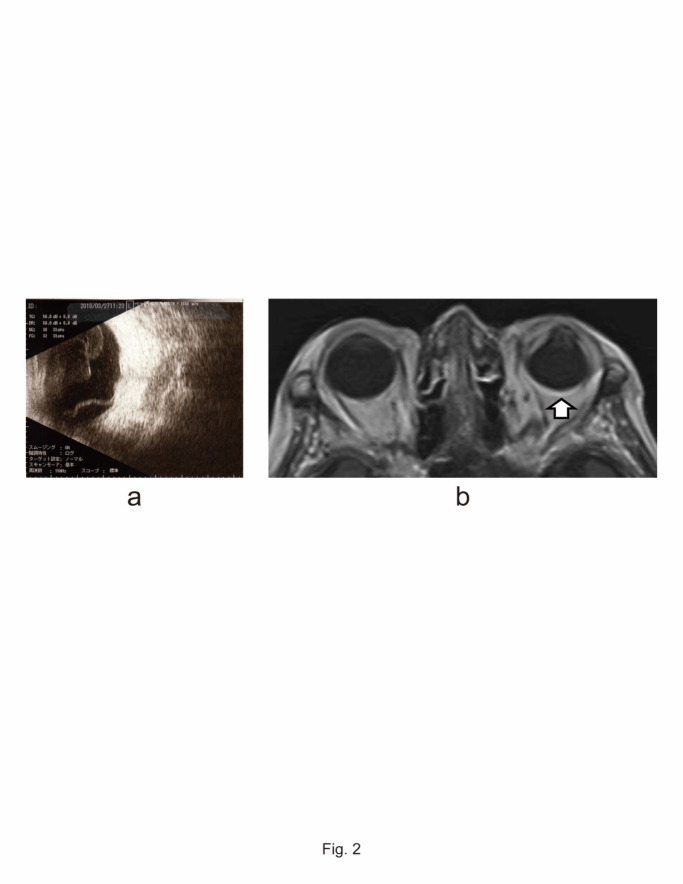
Evident hyperintensity around the left ocular globe including posterior sclera. (a) B mode-echo shows retinal detachment and shadow of mass in the vitreous; (b) Brain and orbit magnetic resonance imaging with gadolinium

A 79-year-old woman underwent primary pterygium excision and conjunctival autograft transportation with mitomycin C (MMC) in her left eye in June 2016. One year and 6 months postoperatively, she complained of minor congestion at the excision site. Topical fluoroquinolone and fluorometholone 4 times a day were prescribed. Three months later, she presented with redness and pain in her left eye. Diffuse anterior scleritis with multiple peripheral corneal infiltrates was noted; however, scleral necrosis was not apparent (**Fig. 1a, b**). Neither flare nor cells were observed in the anterior chamber. Fundus observation was not possible due to a cataract and posterior synechia. B-mode echo revealed intravitreal shadow and retinal detachment. Magnetic resonance imaging with gadolinium showed hyperintensity around the left ocular globe including the posterior sclera, indicating an inflammatory reaction (**Fig. 2a, b**). Intraocular pressure was normal at 14 mmHg.

The patient had been aware of severe eye pain for several weeks; however, the pain was relatively mild at the visit. As she had no light perception in her left eye and with her advanced age, we did not initiate systemic immunosuppressive treatment. Instead, she was treated with topical 0.1% betamethasone and 0.5% levofloxacin. 

The scleritis remained active three months after the initiation of treatment. Multiple granular deposits were observed on the surface of the iris (**Fig. 1c**). The deposits gradually increased over the next 4 weeks, and some had detached from the iris and were floating in the anterior chamber (**Fig. 1d, e**). The deposits, aqueous humor, and vitreous were pathologically examined. Vitreous samples were histologically negative for malignant cells, and culture revealed no growth of bacteria or fungi. The IL10:IL6 ratio in the vitreous was low at 0.36, essentially ruling out malignant lymphoma. The samples from the vitreous and anterior chamber were negative for bacteria, fungi, herpes simplex virus, and varicella-zoster virus DNA using real-time PCR. Microscopic examination revealed that the deposits were inflammatory granulomas, but not bacterial masses or malignant tissue.

The patient was referred to the Department of Immunology and Collagen Disease at Tottori University. Although serological examination indicated that antineutrophil cytoplasmic antibody (ANCA) with p-type was positive, diagnosis of systemic collagen disease or vasculitis was not definitively established. Since there were no signs of infection or malignancy, the patient was diagnosed with SINS with intraocular inflammation following pterygium excision. We presumed that the deposits on the iris were nonspecific findings caused by chronic progression of intense intraocular inflammation. Even though aggressive treatment was not initiated, the intensity of the ocular inflammation, redness, and pain gradually declined (**Fig. 1f, g**). The eyeball became atrophic with diffuse thinning of the anterior sclera, and the deposits on the iris surface almost completely disappeared.

The study protocol conformed to the tenets of the Declaration of Helsinki and was approved by the Ethics Review Committee of Nojima Hospital. Written informed consent was obtained from the patient.

## Discussion

The duration of the latent period between surgery and the onset of SINS varied from the first operative day to several years [**1**-**3**,**5**,**7**,**8**]. In our patient, scleritis occurred 1 year and 6 months following pterygium surgery. O’Donoghue et al. reviewed the clinical features of 52 eyes from 43 patients who developed scleritis following ophthalmic surgery. Necrotizing anterior scleritis, SINS, was identified in most cases (94%), and 11 cases (23%) had evidence of secondary posterior scleritis. Three-fifths of the cases had a history of some systemic disorder. This review did not include a single case associated with pterygium surgery [**3**]. A possible explanation for the few reports of SINS following pterygium surgery is that the scleritis or necrosis may not have been diagnosed as SINS. Instead, they may have been diagnosed as necrosis due to other mechanisms. While the etiology of SINS is unclear, some researchers have hypothesized that the condition represents a hypersensitivity reaction. Deposition of the immune complex in the scleral vessels may be stimulated by surgical trauma. However, scleral necrosis and melting can occur following pterygium surgery from the use of MMC, infection, adjunctive irradiation, or by excessive cauterization of the sclera [**4**,**9**]. 

Lu et al. retrospectively assessed treatment of 9 cases of scleral necrosis following pterygium excision [**4**]. None of the potential causes of scleritis, such as MMC application, irradiation, or infection, were present; therefore, a diagnosis of SINS was proposed. In this report, 4 of the 9 patients were diagnosed with SINS and were treated with immunosuppressive therapy.

In the review of literature, more than half of SINS cases are associated with systemic disease, [**3**], while patients with SINS who undergo pterygium surgery have rarely exhibited a history of systemic disease [**4**-**8**]. If the etiology of SINS is hypersensitivity to an immune complex from systemic disease or a previous history of ocular surgery, and is common to pterygium and other surgeries, there should be no difference between pterygium and other cases. It is likely that cases diagnosed with SINS following pterygium surgery include necrosis from causes other than those for SINS. Accompanying necrosis at the site of the excision can be found in most cases of SINS following pterygium excision. Postoperative invasion or focal hypersensitivity should be most evident at the surgical site. The adverse effects of MMC application, infection, and radiation should also be most evident at the surgical site [**4**-**9**]. It is not possible to definitively determine whether scleritis that has developed following pterygium excision is due to surgically induced hypersensitivity.

## Conclusion

In our case, scleral necrosis of the surgical site was not significant; however, we ultimately observed a wide area of necrosis, suggesting that the etiology of our case of SINS was immunological. To our knowledge, this is the first report of anterior diffuse scleritis accompanied by posterior scleritis following primary pterygium excision.

**Conflict of interest**

Authors state no conflict of interest.

**Informed Consent and Human and Animal Rights statements**

Informed consent has been obtained from all individuals included in this study.

**Authorization for the use of human subjects:**

Ethical approval: The research related to human use complies with all the relevant national regulations, institutional policies, is in accordance with the tenets of the Helsinki Declaration, and has been approved by the Ethics Committee of Nojima Hospital, Tottori, Japan.

**Acknowledgements**

None.

**Sources of Funding**

None.

**Disclosures**

None.
